# Editorial: Artificial intelligence in process modelling in oncology

**DOI:** 10.3389/fonc.2023.1298446

**Published:** 2023-12-12

**Authors:** Carlos Fernandez-Llatas, Roberto Gatta, Fernando Seoane, Vincenzo Valentini

**Affiliations:** ^1^ ITACA-SABIEN Technologies for Health and Well-Being, Polytechnic University of Valencia, Valencia, Spain; ^2^ Department of Clinical and Experimental Sciences, University of Brescia, Brescia, Italy; ^3^ Department of Clinical Science, Intervention and Technology, Karolinska Institutet (KI), Stockholm, Sweden; ^4^ Department of Textile Technology, Faculty of Textiles, Engineering and Business, University of Borås, Borås, Sweden; ^5^ Department of Clinical Physiology, Karolinska University Hospital, Stockholm, Sweden; ^6^ Department of Medical Technology, Karolinska University Hospital, Huddinge, Sweden; ^7^ Agostino Gemelli University Polyclinic (IRCCS), Rome, Italy; ^8^ Catholic University of the Sacred Heart, Rome, Italy

**Keywords:** process mining, process modelling, artificial intelligence, pattern of care, clinical processes

## 1 Introduction

From the early days of process mining in healthcare, oncology has consistently been one of the most compelling application areas (Rojas et al. (1)). The various oncological diseases, in fact, from the perspective of screening, diagnosis, treatment, and follow-up pathways, although sharing some common elements, also differ considerably depending on the anatomical district involved, while maintaining a generally well-structured practice, codified in consensus, protocols, and guidelines. In addition, the significant impact on patients and the high social costs contribute in making this sector one in which Data Analysis seeks to provide a meaningful contribution through the application of innovative techniques. Since in this context, it is particularly important to capture the temporal evolution of relevant factors, the application of Artificial Intelligence (AI) techniques for modeling clinical/healthcare processes can be a key tool in understanding what may play a significant role in disease control or the induction of iatrogenic events in the care pathway.

In this Research Topic, many applications covering different areas of Process-Modelling in the oncology theme have been explored.

In Tozzi et al., by a systematic review of systematic reviews, an exploration of the current contributes of AI in Pediatric Oncology is given, in Europe in particular. A set of 34 reviews and 304 articles were considered, retrieved by querying the Web of Science platform. The number of original papers, relatively stable from 2004 to 2016, quadruples in 2018 and subsequently doubles compared to 2018 in 2020. This is interpreted as a sign of the growing interest in paradigms, methods and tools provided by AI and how, nowadays, it is seen as promising in the field of oncology. Notably, despite the considerable amount of retrieved papers, there was no evidence found for AI utilization in process mining, clinical pathway modeling, or computer-interpreted guidelines to enhance healthcare processes. This indicates that AI in oncology is probably just beginning, and there is room for various tools, like those focused on process analysis, for proposing efficient solutions for many unmet needs in pediatric oncology.

More focused contributions showcasing specific benefits of AI-based process modeling are present in this Research Topic.

In Cuendet et al. the authors demonstrate how Process Discovery algorithms can easily and effectively visualize the pathways of oncology patients (in the acute phase of the disease) during the COVID pandemic, evaluating the differences between these and non-oncology patients (in a non-acute phase) in both waves. Here they propose an innovative approach, called Differential Process Mining, mixing common statistical tests and a Process Discovery algorithm, able to statistically measure the differences in terms of life expectancy and variations in access times to various healthcare facilities.

The use of conformance checking techniques, on the other hand, is present in Savino et al. Here, PWL Computer Interpretable Clinical Guideline (CICG) language, is used on a cohort of patients affected by rectal cancer to measure the adherence with the Clinical Practice Guidelines by ESMO (European Society of Medical Oncology). Interestingly, while the distribution of non-compliant patients across four risk categories was notably high, the clinical outcomes were comparable or even slightly better and this has been recognized as being related to the fact that some procedures have been updated with more recent evidence. This result opens up the interesting prospect in which Process Mining may also propose algorithms capable of autonomously improving an incoming clinical guideline with new clinical evidence (a domain-specific adaptation of the so called Process Enhancement).

Another practical application is shown in Wicky et al. Here a process is discovered starting from the paths of a cohort of 303 patients affected by advanced melanoma and treated with Imunotherapy, Radiotehrapy and Chemotherapy. The authors propose a significant evolution of careflow mining algorithm They Dagliati et al. (2) in which at each node of the graph, it is possible to observe a Kaplan Meier survival curve related to the patients passing through that node. The graph is updated daily and integrated into the software tool that doctors have access to for research purposes. This dissemination strategy is aimed at enhancing the data analysis activities, suggesting new exploratory hypotheses, and significantly broaden the audience of researchers able to benefit from these innovative techniques.

Similarly, aiming at making clinical data more accessible to support cancer health professionals in analysing processes, Valero-Ramon et al. shows and exploratory analytical Interactive Process Mining tool, integrated within a daily used Dashboard for monitoring cancer patients’ pathways. The tool is a part of a more general proposed methodological framework, designed to support interactive and iterative iterations among physicians and Data Scientists and can be easily applied on all the cancer type. The tool is rich of features and provide tools for data inspections, querying, clustering, process discovery, presenting understandable results visually and navigable.

In Kalendralis et al. is shown how AI can be also used to cope in trying to assure the quality of radiotherapy treatment plans. In this contribute, the authors extend the analysis in a multi-centric perspective. Here a Bayesian network is trained to identify the risk to expose the patients tu sub-optimal treatment plans collecting the experience of three hospitals, in United States and Europe.

**Figure 1:**
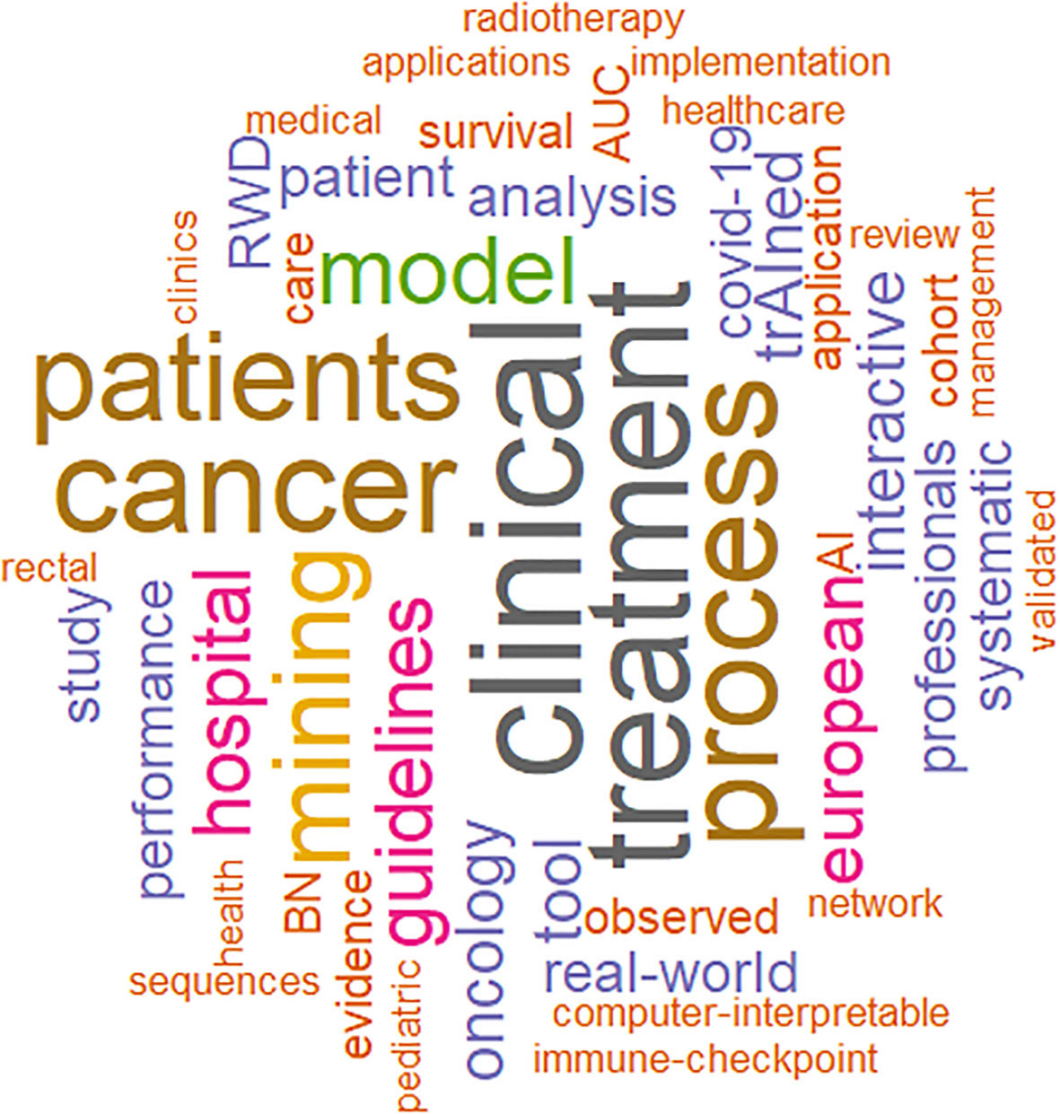
Word cloud of terms used in the abstracts of articles in the Research Topic.

From this Research Topic (**Figure 1** shows the most significant terms in this article collection) and the underlying bibliography, clear signals emerge: on one hand, there is an increasing need to address the growing accumulation of data collected from clinical practice, which is becoming more heterogeneous and structured. On the other hand, process analysis using AI-based techniques, although still in its early stages of practical application, appears to possess innovative tools compared to what is currently available. This gap now represents the greatest opportunity, as recognized in the manifesto of Process Mining for Healthcare Munoz-Gama et al. (3), that unites the international alliance and aims to bring out the true potential of this approach by focusing on the practical application of disciplines related to real-world data.

## Author contributions

CF-L: Writing – original draft, Writing – review & editing. RG: Writing – original draft, Writing – review & editing. FS: Writing – original draft, Writing – review & editing. VV: Writing – original draft, Writing – review & editing.
